# Effects of disease activity on lipoprotein levels in patients with early arthritis: can oxidized LDL cholesterol explain the lipid paradox theory?

**DOI:** 10.1186/s13075-020-02307-8

**Published:** 2020-09-11

**Authors:** Ana M. Fernández-Ortiz, Ana M. Ortiz, Silvia Pérez, Esther Toledano, Lydia Abásolo, Miguel A. González-Gay, Santos Castañeda, Isidoro González-Álvaro

**Affiliations:** 1 Rheumatology Unit, Hospital General de Almansa, Albacete, Spain; 2 Rheumatology Division, Hospital Universitario La Princesa, IIS-IP, Diego de León 62, 28006 Madrid, Spain; 3 Rheumatology Division, Hospital Universitario Fundación Jiménez Díaz, Madrid, Spain; 4 Rheumatology Division, Hospital Clínico San Carlos, IdISSC, Madrid, Spain; 5 Rheumatology Division, Hospital Universitario Marqués de Valdecilla, IDIVAL, University of Cantabria, Santander, Spain; 6 Cátedra UAM-Roche, EPID-Future, Universidad Autónoma Madrid, Madrid, Spain

**Keywords:** Rheumatoid arthritis, Cholesterol, oxLDL-C

## Abstract

**Background:**

An increased risk of cardiovascular (CV) complications has been described in patients with rheumatoid arthritis (RA). It is the result of the combined effect of classic CV risk factors and others that are specific to the disease.

**Methods:**

We assessed data from 448 early arthritis (EA) patients: 79% women, age (median [p25-p75]) at onset: 55 [44–67] years and disease duration at study entry 5 [3–8] months; and 72% fulfilled the 1987 RA criteria at 2 years of follow-up. Rheumatoid factor was positive in 54% of patients and anti-citrullinated peptide antibodies in 50%. The follow-up of patients ranged from 2 to 5 years with more than 1400 visits with lipoprotein measurements available (mean 2.5 visits/patient). Demographic- and disease-related variables were systematically recorded. Total cholesterol (TC), high-density lipoprotein (HDL-C), and low-density lipoprotein (LDL-C) levels were obtained from routine laboratory tests. Oxidized-LDL (oxLDL-C) levels were assessed using a commercial ELISA kit. We fitted population-averaged models nested by patient and visit to determine the effect of independent variables on serum levels of TC, its fractions, and oxLDL-C.

**Results:**

After adjustment for several confounders, high-disease activity was significantly associated with decreased TC, HDL-C, and LDL-C levels and increased oxLDL-C levels. Standardized coefficients showed that the effect of disease activity was greater on oxLDL-C and HDL-C. Interestingly, we observed that those patients with lower levels of LDL-C showed higher oxLDL-C/LDL-C ratios.

**Conclusions:**

High-disease activity in EA patients results in changes in the HDL-C and oxLDL-C levels, which in turn may contribute to the increased risk of CV disease observed in these patients.

## Background

There is a great body of evidence supporting that patients with rheumatoid arthritis (RA) display a higher risk of cardiovascular events (CVE) than the general population [1–5]. In addition, several studies have shown that scores for predicting cardiovascular (CV) risk in the general population based on traditional CV risk factors (smoking, hypertension, obesity, diabetes, and dyslipidemia) underestimate the CV risk in patients with RA [6–10]. This additional risk for CVE has been studied in terms of specific genetic background [3, 11] and in relation with the effect of disease activity on lipid profile in these patients [12–19].

There is an increased CV risk in the general population associated with an increase in total cholesterol (TC), mainly low-density lipoprotein cholesterol (LDL-C), and a decrease in high-density lipoprotein cholesterol (HDL-C) levels, and, as a consequence, an increase in the atherogenic index [20]. However, in patients with active RA and other inflammatory disorders a “paradoxical phenomenon” occurs with a decrease in TC and LDL-C associated with an increase in CV risk [5, 21, 22]. Furthermore, recently, it has been observed that those patients with very low LDL-C show a stronger association with subclinical coronary atherosclerosis, similar to that of patients with the highest levels of LDL-C, which has been termed “the lipid paradox theory” [23]. The relationship between changes in the lipid metabolism and RA disease activity has been analyzed in previous studies with controversial results [12–19]. Several concerns related with previous studies may explain these controversial results. Lipid profile in RA has been studied both in observational cross-sectional studies of long-standing RA and in randomized clinical trials in patients with early arthritis (EA). Moreover, many of these studies included a low number of patients and different outcomes were measured in each work [12–19, 24–26].

An issue of potential interest regarding lipid profile disturbances that appear over time in patients with RA is whether these alterations are observed at the early stages of the disease. To address this question, we analyzed how disease activity affects the lipid profile in a population of patients with early arthritis without any treatment at the baseline visit, who were followed-up for up to 5 years in five protocolized visits.

## Patients and methods

### Design and patients

Retrospective analysis of data collected in the PEARL (Princesa Early Arthritis Register Longitudinal) study in order to determine the effect of disease activity on the lipid profile of patients with early arthritis.

PEARL study includes incident cases of patients with one or more swollen joints for less than a year referred to our EA Clinic. Patients with gout, septic or viral arthritis, osteoarthritis, spondyloarthritis, or connective tissue diseases diagnosed during the follow-up period were excluded from this analysis. Only those patients fulfilling the 1987 ACR criteria for RA classification [27] and those considered undifferentiated arthritis (UA) [28] after 24 months of follow-up were included in this study. The register includes 5 structured visits (baseline, 6, 12, 24, and 60 months) in which socio-demographic, clinical, laboratory, therapeutic, and radiological data as well as biological samples are systematically collected in parallel with routine analysis after, at least, 10 h of fasting.

It is important to point out that there is no pre-established therapeutic protocol in the PEARL study, so the decision on when and how to treat the patients during the follow-up relies on the responsible physicians from the rheumatology department (see Supplementary Table 1 for the description of treatments along with the follow-up). Nevertheless, the register specific evaluation visits are performed by only two rheumatologists (AMO, IG-A) in an attempt to achieve more accurate clinical evaluation, especially regarding joint counts. A more detailed description of the PEARL study has been previously published [29]. The register started in 2000 and it is still ongoing. However, for this analysis, we only used the visits included in the database until June 2018.

Disease activity stratification was performed applying cut-offs previously described: DAS28 estimated using either erythrocyte sedimentation rate (ESR) [30] or C-reactive protein (CRP) [31], Simplified Disease Activity Index (SDAI) and Clinical Disease Activity Index (CDAI) [32], and Hospital Universitario Princesa Index (HUPI) [33]. Disability was assessed with the Spanish version of the Health Assessment Questionnaire (HAQ) [34].

### Lipid profile measurements

TC levels, HDL-C, LDL-C, very low-density lipoproteins (VLDL-C) fractions, and triglycerides (TG) levels were estimated in routine blood tests at the local biochemistry laboratory of our center. TC, HDL-C, and TG concentrations were determined through the enzymatic method in a Hitachi 911 analyzer (CHOP-PAP, GPO-PAP, and HDL-direct, respectively; Roche Diagnostics). LDL-C was estimated through the Friedewald formula [35, 36]: LDL = TC−HDL–0.2 × TG.

Although it was established per protocol to measure the total lipid profile in all visits, due to the fact that some patients missed some visits or to the occasional decision of the biochemistry laboratory to skip measuring cholesterol fractions when TC was below 200 mg/dl, there were missing data for some of the variables. Therefore, the data from blood tests available for analyses were TC from 1416 visits, HDL-C from 977 visits, LDL-C from 929 visits, VLDL-C from 717 visits, and TG from 848 visits.

### Oxidized-LDL evaluation

Since our center does not routinely measure oxidized-LDL-C (oxLDL-C) levels, we determined them in frozen serum samples from our register through a commercial ELISA kit (Mercodia AB, Uppsala, Sweden) following the manufacturer’s instructions for use. To evaluate the effect of frozen storage time on oxLDL-C measurements, we analyzed a subpopulation of 104 samples with a range of storage from 6 to 40 months evenly distributed throughout our register. As it is shown in Supplementary Figure 1, for periods of frozen storage longer than 12 months, the capability to measure oxLDL-C levels was severely impaired. Therefore, we decided to perform the current study exclusively with samples that were frozen for less than 12 months. This approach allowed us to obtain data about oxLDL-C serum levels from 270 visits from a subpopulation of 167 patients (1.5 visits per patient). The intra- and inter-assay variability were calculated by repeating four samples (two with high value and two with a low value) in six wells of four independent ELISA plates. According to our measurements, intra-assay and inter-assay variability coefficients were 9.5% and 22.3%, respectively.

### Statistical analysis

Normally distributed quantitative variables were represented as the mean (± standard deviation: SD), while non-normally distributed variables were represented as the median and interquartile range (IQR). Qualitative variables were described using a calculation of the proportions. Variables with a normal distribution were analyzed by the *t* test or ANOVA, while the Mann-Whitney or Kruskal-Wallis tests were used for variables with a non-normal distribution. A *χ*^2^ or Fisher’s exact test was used to compare categorical variables.

After bivariate analysis to identify independent factors that influenced TC, HDL-C, LDL-C, oxLDL-C, VLDL-C, and TG levels during the follow-up, we fitted six population-averaged models by generalized linear models nested by the patient and visit using the *xtgee* command of Stata 12.1 for Windows (StataCorp LP, College Station, Texas, USA). Since oxLDL-C values did not follow a Gaussian distribution, this variable was normalized by calculating the square root of its values. In this case, the sqrt_oxLDL-C variable was used as a dependent variable. The remaining models were fitted with the raw data since TC, HDL-C, LDL-C, VLDL-C, and TG levels approximately followed Gaussian distributions. The population-averaged generalized estimating equations were first modeled by adding all variables with a *p* value < 0.15 in the bivariate analysis. The final models were constructed using quasi-likelihood estimation based on the independence model information criterion [37] and Wald tests, removing all variables with *p* > 0.15. The variable time of frozen storage was always included in the multivariate analysis of oxLDL-C levels. Since six different multivariate analyses were fitted, to adjust for multiple comparisons, we applied Bonferroni correction and the statistical significance was set at < 0.01.

We performed sensitivity analyses for all dependent variables repeating the statistical models only with the visits in which patients were not taking statins. These sensitivity analyses yielded no relevant differences with the models that included the whole population (Supplementary Table 2).

In addition, to compare which variables had a proportionally higher influence on the lipoprotein profile, we estimated standardized coefficients by repeating the multivariate analysis with new standardized variables generated with the egen command of Stata with the option *std*, which produces variables with mean 0 and standard deviation 1.

To determine which component of disease activity indexes was responsible for most of the effect on lipids, in the models with standardized variables, we replace the variable disease activity by the individual standardized components, namely CRP, swollen and tender joint counts, and global disease assessment by the patient.

## Results

### Description of the population

The baseline characteristics of patients are shown in Table 1. Briefly, the study sample comprised 448 patients; 79% of them were women and 324 (72%) fulfilled 1987 ACR criteria for RA classification, while 124 (28%) were classified as UA. The median age at baseline was 55 years (interquartile range [IQR] 44–67) and the median disease duration 5 months (IQR: 3–8). Baseline disease activity on average was moderate with all the 5 indexes used in the study.

**Table 1:** Baseline characteristics of patients included in the PEARL study

	Total population (*N* = 448)	oxLDL not studied (*N* = 281)	oxLDL studied (*N* = 167)	*p*
Female gender (%)	354 (79)	224 (79.7)	130 (77.8)	0.638
Age (years)	55 [44–67]	58 [44–68]	52 [43–65]	0.08
Disease duration (months)	5 [3–8]	5 [3–8]	6 [4–9]	**0.004**
DAS28	4.4 [3.3–5.5]	4.1 [3.3–5.5]	4.7 [3.5–5.7]	0.060
SDAI	18 [9.6–29.2]	17 [8.8–29]	19.2 [10.4–29.5]	0.183
HUPI	7 [4.5–10]	7 [4–10]	8 [5–9.5]	0.174
HAQ	0.875	0.875	1	0.059
	[0.5–1.625]	[0.375–1.625]	[0.625–1.625]	
RF (%)	54	57	48	0.054
ACPA (%)	50	53	46	0.175
UA (%)	28	26	30	0.297
Smoking (%)	21.4	20	24.2	0.36
Statins use (%)	10.7	12	9	0.361
BMI p50 [IQR]	26.1 [23.4–29]	25.9 [22.9–29]	26.3 [23.9–29.1]	0.171

Significance was considered if *p* <  0.05. **In bold** significant *p* values

Abbreviations: *ACPA* anti-citrullinated protein antibodies, *BMI* body mass index, *DAS28* Disease Activity Score (28 joints count), *HAQ* Health Assessment Questionnaire, *HUPI* Hospital Universitario La Princesa Index, *IQR* interquartile range, *N* number, *oxLDL* oxidized LDL cholesterol, *p p* value, *PEARL* Princesa Early Arthritis Register Longitudinal, *RF* rheumatoid factor, *SDAI* Simplified Disease Activity Index, *UA* undifferentiated arthritis

The HAQ showed mild/moderate disability. Almost half of the population suffered from the seropositive disease [(rheumatoid factor (RF) or anti-citrullinated peptide antibodies (ACPA)], and 21% were active smokers and the average body mass index (BMI) of patients indicated a slight overweight. Less than 11% of patients were using statins at baseline and information on the use of these drugs was collected at each visit.

### Differences between patients with or without oxLDL-C measurement

As shown in Table 1, oxLDL-C was assessed in 167 of the 448 patients. This subgroup of patients had longer disease duration at baseline and a non-significantly increased disease severity. However, other features were similar in both groups. (Table 1). Therefore, patients in which oxLDL-C was analyzed constituted a representative cohort of Spanish individuals with EA. In this regard, Supplementary Table 1 shows that patients were mainly treated with methotrexate, antimalarials, and leflunomide without significant differences between both groups. In addition, a non-significant trend to higher percentages of patients treated with tocilizumab, abatacept, or rituximab was observed in patient in which oxLDL-C measurement was not carried out.

### Variables associated with TC levels throughout the follow-up of early arthritis patients

As shown in Table 2, TC was significantly higher in female and patients older than 45 years, as well as in those visits in which patients were being treated with leflunomide. In addition, a non-significant trend to higher levels of TC was observed with increasing BMI, as well as in visits in which the patients were being treated with abatacept (Table 2). As expected, TC was significantly lower in those visits in which patients were being treated with statins (Table 2).

**Table 2:** Variables that have an influence on lipid profile of patients with early arthritis

	Total cholesterol (mg/dl)		HDL cholesterol (mg/dl)		LDL cholesterol (mg/dl)		oxLDL (sqrt U/ml)	
	*β* coeff. ± s.e.	*p*	*β* coeff. ± s.e.	*p*	*β* coeff. ± s.e.	*p*	*β* coeff. ± s.e.	*p*
Gender								
Male	Ref.	–	Ref.	–	n.i.	–	Ref.	–
Female	10.5 ± 3.4	**0.002**	12.4 ± 1.9	**< 0.001**			−1.03 ± 0.48	0.031
Age								
< 45 years	Ref.	–	Ref.	–	Ref.	–	n.i.	
45–65 years	23.1 ± 3.3	**< 0.001**	1.9 ± 1.9	0.319	16.4 ± 3.3	**< 0.001**		
> 65 years	23.9 ± 3.7	**< 0.001**	5.6 ± 2.1	**0.008**	12.3 ± 3.7	**0.001**		
BMI	0.68 ± 0.29	0.019	−0.62 ± 0.16	**< 0.001**	0.71 ± 0.29	0.016	n.i.	
DA								
Remission	Ref.	–	Ref.	–	Ref.	–	Ref.	–
Low	2.19 ± 1.74	0.582	−0.79 ± 1.04	0.442	1.96 ± 2.13	0.359	0.28 ± 0.48	0.564
Moderate	−3.04 ± 1.90	0.110	−3.30 ± 1.14	**0.004**	1.35 ± 2.30	0.556	0.43 ± 0.48	0.367
High	−14.95 ± 2.53	**< 0.001**	−8.89 ± 1.56	**< 0.001**	−6.39 ± 2.92	0.029	1.88 ± 0.66	**0.004**
Statins	−27.4 ± 3.05	**< 0.001**	n.i.		− 28.7 ± 3.2	**< 0.001**	n.i.	
Smoking	n.i.		n.i.		7.0 ± 4.5	0.118	n.i.	
MTX (mg/wk)	n.i.		2.06 ± 0.94	0.029	n.i.		n.i.	
Leflunomide (mg/d)	6.4 ± 2.2	**0.003**	2.35 ± 1.27	0.064	4.79 ± 2.57	0.062	n.i.	
Abatacept (Y/N)	36.94 ± 16.01	0.021	40.54 ± 9.57	**< 0.001**	n.i.		n.i.	
TNF i (Y/N)	n.i.		n.i.		n.i.		1.597 ± 1.003	0.111
LDL (mg/dl)	n.i.		n.i.		n.i.		0.015 ± 0.005	**0.005**
Frozen storage (months)	n.i.		n.i.		n.i.		−0.003 ± 0.001	**< 0.001**

Abbreviations: *β coeff.* beta coefficient, *BMI* body mass index, *DA* Disease activity assessed with, *HUPI* Hospital Universitario La Princesa Index, *HDL* high-density lipoproteins, *LDL* low-density lipoproteins, *MTX* methotrexate, *N* no, *n.i.* not included (not relevant to the model), *oxLDL* oxidized LDL, *p p* value, *Ref.* reference variable, *s.e.* standard error, *sqrt* square root, *n.i.* not included (not relevant to the model), *TNFi* TNF inhibitors, *wk* week, *Y* yes. Due to multiple comparisons, statistical significance was considered if *p* < 0.01. **In bold,** significant *p* values

After adjustment for these confounders, we observed that only high-disease activity according to HUPI assessment was significantly associated with lower levels of TC (Table 2).

**Fig. 1:**
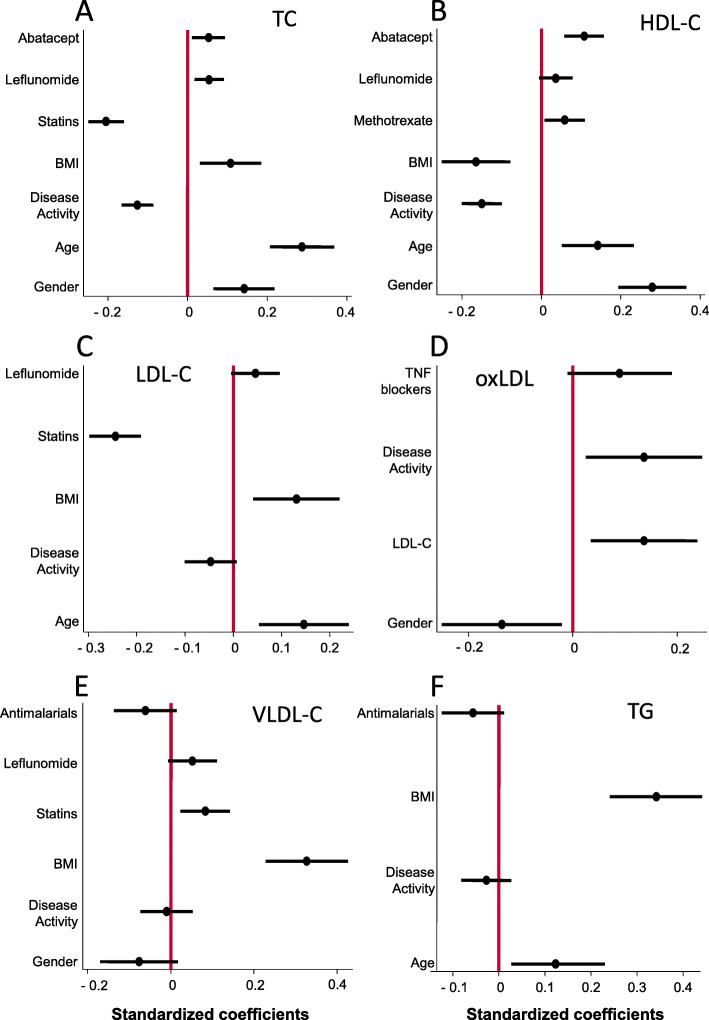
Effect of different variables on the lipid profile of patients with early arthritis. **a** Total cholesterol. **b** HDL cholesterol. **c** LDL cholesterol. **d** Oxidized LDL cholesterol. **e** VLDL cholesterol. **f** Triglycerides. Data are shown as the respective standardized coefficients (black dot) and their 95% confidence interval (black bar) estimated with the multivariate analysis described in the “Patients and methods” section

The most relevant variable affecting TC was age, followed by statins (Fig. 1a). Disease activity had an effect similar to those of gender and BMI (Fig. 1a).

Similar findings were observed when disease activity was assessed with other indexes (Supplementary Tables 3, 4, 5, and 6).

### Variables associated with HDL-C levels throughout the follow-up

As described for TC, female patients showed a significant increase of HDL-C levels compared to male patients (Table 2). Using individuals younger than 45 years as a reference, a trend for high HDL-C levels was observed in patients older than 65 years (Table 2). In contrast, HDL-C levels significantly decreased with increasing values of BMI (Table 2). After adjustment for these variables, the multivariate analysis showed that increasing levels of disease activity induced a dose-dependent decrease of HDL-C, being statistically significant for moderate and high-disease activity (Table 2). Treatment with methotrexate, leflunomide, and abatacept was also associated with an independent increase of HDL-C levels, which was only statistically significant for abatacept (Table 2).

Regarding the magnitude of the effect on HDL-C levels, disease activity showed an effect size similar to BMI, age, and gender, whereas the relative effect of treatments seemed to be lower (Fig. 1b). Additional information about data related to disease activity assessed with other indexes is shown in Supplementary Tables 3, 4, 5, and 6.

### Variables associated with LDL-C levels throughout the follow-up

As expected, LDL-C levels were significantly lower in those visits in which the patients were being treated with statins (Table 2). In contrast, LDL-C levels were significantly higher in patients older than 45 years and increased in parallel with increasing BMI (Table 2).

High-disease activity was significantly associated with lower LDL-C levels (Table 2), although compared to other variables that significantly affected LDL-C levels, the effect of disease activity was clearly mild (Fig. 1c). Similar findings were observed when disease activity was assessed with other activity indexes (Supplementary Tables 3, 4, 5, and 6).

### Variables associated with oxidized LDL-C levels

As mentioned in the “Patients and methods” section, the frozen time of serum samples significantly decreased the capability to detect oxLDL-C (Table 2 and Supplementary Figure 1). In addition, oxLDL-C levels were directly associated with total LDL-C levels (Table 2). Women showed slightly lower oxLDL levels than men, although no statistical significance was reached (Table 2).

After adjustment for these relevant variables, the multivariate analysis showed a significant increase of oxLDL-C levels when disease activity was high (Table 2). This effect was similar to that of LDL-C levels (Fig. 1d).

Additional information about data related to disease activity assessed with other indexes is shown in Supplementary Tables 3, 4, 5, and 6.

**Fig. 2:**
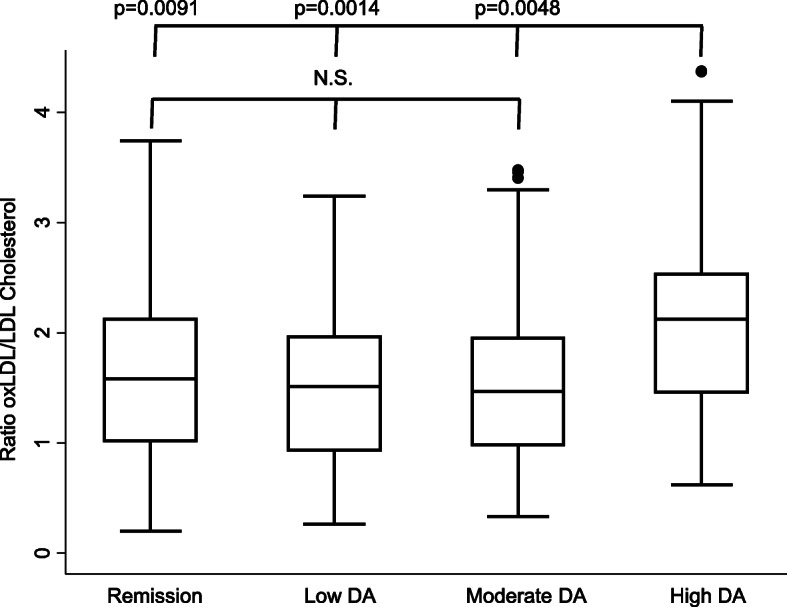
Effect of disease activity on the ratio of oxidized LDL cholesterol to total LDL cholesterol of patients with early arthritis. Data are represented as box plots including median (line inside the boxes) and the percentiles 25, 75 (lower and upper lines of the boxes), and 10 and 90 (endpoints of the lines outside the boxes). Dots represent outliers. The level of disease activity was established with HUPI as previously described [33]. Statistical significance was established with the Mann-Whitney test. Due to multiple comparisons, the significance level was set to 0.008

**Fig. 3:**
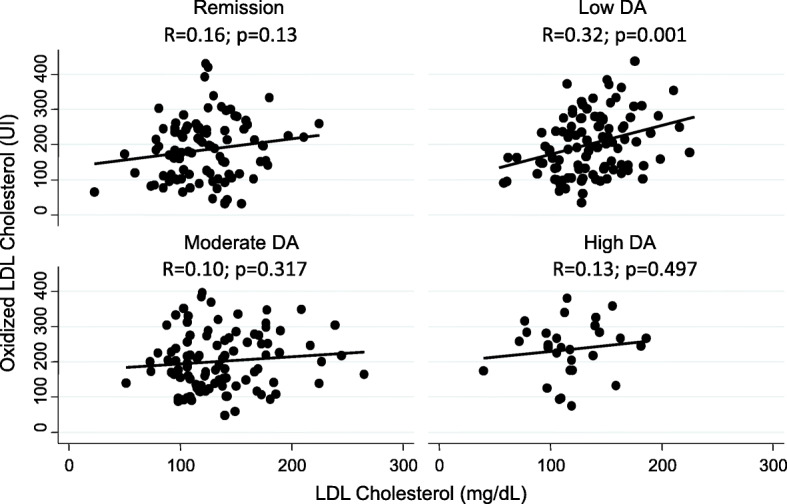
Correlation between oxidized LDL cholesterol and total LDL cholesterol according to the level of disease activity in patients with early arthritis. Data are shown as dot plots of the values in the visits in which the patients were in remission (upper left panel), low (upper right panel), moderate (lower left panel), or high-disease activity (DA) established with HUPI as previously described [33]. Black lines represent the respective linear regressions estimated through the *twoway lfit* command of Stata 12.1. *R* and *p* values were obtained with the Pearson’s test

**Fig. 4:**
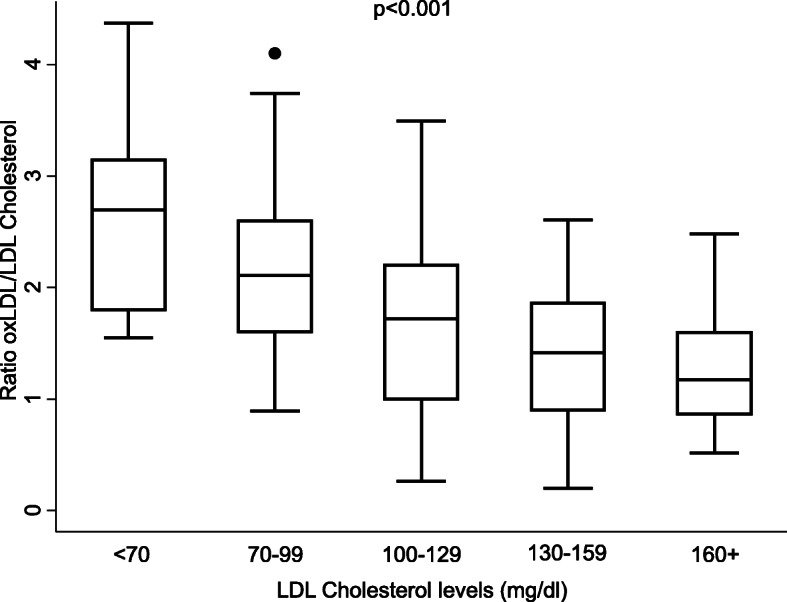
Ratio of oxidized LDL cholesterol to LDL cholesterol in patients with early arthritis classified in different strata according to their LDL cholesterol level. Data are represented as box plots including median (line inside the boxes) and the percentiles 25, 75 (lower and upper lines of the boxes), and 10 and 90 (endpoints of the lines outside the boxes). Dots represent outliers. Strata of LDL cholesterol levels were defined as described by Giles et al. [23]. Statistical significance was established using Cuzick’s non-parametric trend test, and the significance level was set to 0.05

On the other hand, it has been recently described that patients with low-circulating LDL-C or very high-LDL-C show a stronger association with subclinical coronary atherosclerosis than patients with normal LDL-C [23]. We were interested in knowing whether this lipid paradox in RA patients could be explained by LDL-C oxidation. Figure 2 shows that the proportion of oxidized LDL-C (oxLDL-C/LDL-C ratio) was significantly higher in visits in which patients showed high-disease activity. Despite this observation, a clear correlation between oxLDL-C and LDL-C levels was only observed in visits in which patients showed low-disease activity (Fig. 3 upper right panel). Interestingly, in visits in which patients had moderate or high-disease activity, patients with low LDL-C showed similar levels of oxLDL-C than those with higher LDL-C (Fig. 3 lower panels). Furthermore, the proportion of oxLDL-C was significantly higher in patients with the lowest level of total LDL-C (Fig. 4).

### Variables associated with VLDL-C and TG levels

The main variable associated with these lipid fractions was BMI. Neither relevant nor significant effect of disease activity was observed in the serum levels of these two parameters (Fig. 1e and f; Supplementary Tables 7 and 8).

### Standardized effect of disease activity on lipid profile

**Fig. 5:**
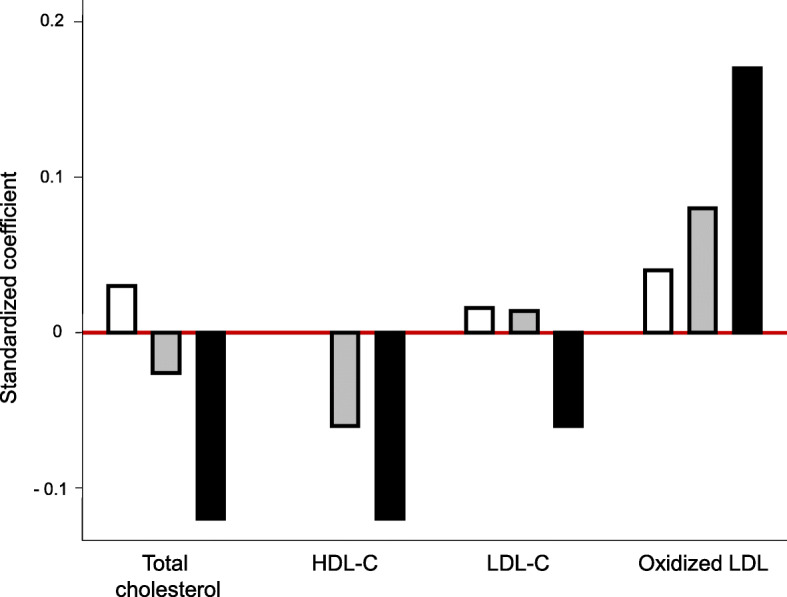
Comparative effect of disease activity on the lipid profile of patients with early arthritis. Data are shown as the standardized coefficients of low-disease activity (DA; white bar), moderate DA (gray bar), and high-disease activity (black bar) from the multivariate analysis described in the “Patients and methods” section. Remission was the reference category of the disease activity variable

Finally, comparing standardized coefficients, Fig. 5 shows that disease activity induced a dose-dependent effect on HDL-C (decreasing its levels) and oxLDL-C (increasing its levels). However, a mild effect on total LDL-C was observed; therefore, the decrease in TC when patients are active mainly relies on the HDL-C fraction (Fig. 5).

On the other hand, we were interested in which component of the composite indexes had a greater influence on the changes in lipids. CRP, followed by the swollen joint count, had the highest effect on lipids, especially on LDL-C, being the effect of tender joints and global disease assessment by the patient which is the lowest (Supplementary Figure 2).

## Discussion

The findings described in this study suggest that high-disease activity in patients with EA induces a transformation of their lipid profile into a pro-atherogenic one by inducing a decrease in HDL-C levels and promoting LDL oxidation. In addition, the longitudinal design of our study and the availability of many variables that may affect cholesterol levels have allowed us to determine that the effect of disease activity on the levels of LDL-C fraction is limited and probably not so relevant. Interestingly, our data show that those patients with low LDL-C suffer a proportionally higher oxidation of this molecule, a finding that may explain the lipid paradox theory.

With regard to the effect of disease activity on lowering HDL-C levels, our data are in accordance with information reported previously [38–40]. Disease activity had a similar effect to other variables, such as gender and BMI, reported to affect HDL-C levels in the general population. However, disease activity has an additional effect on HDL-C since different studies suggest that an inflammatory state not only decreases the levels of HDL-C but also has an influence in its atheroprotective capacity [39, 41, 42]. Therefore, the usual HDL functions are abolished transforming HDL-C into a pro-atherogenic factor [43, 44]. Regarding the mechanisms underlying these changes in the HDL-C fraction, it has been proposed that myeloperoxidase (MPO) released by phagocytes during the inflammatory process can induce the production of substances driving changes in the HDL-C fraction, especially oxidation of lipids and apoprotein A1 (apoA1) [45].

Our data also shed light into the paradoxical finding of low LDL-C levels in RA together with a high prevalence of CV disease associated with this disorder. Our multivariate analysis let us unravel that disease activity has an effect on total LDL-C levels in patients with EA. Among the different components included in disease activity indexes, CRP displayed the highest effect in lowering TC and LDL-C. Thus, our results point to IL-6 as a responsible for this finding, since it has been described that this cytokine increases the LDL receptor on hepatocytes and LDL-C catabolism, that improve after blockade of IL-6 signaling [46–48]. However, the inflammatory status, especially in patients with high-disease activity, has the most potent association with LDL oxidation compared to its effect in other lipid fractions. This observation is in accordance with previous reports [49]. In addition, the patients with lower levels of LDL-C were those with proportionally higher levels of oxLDL-C, which could explain why these patients show higher levels of atherosclerosis [23]. Furthermore, it has been described as an increase in anti-oxLDL antibody levels when RA patients are active which improves after controlling the disease activity, suggesting that oxLDL-C may act as an autoantigen [50].

Regarding the mechanisms underlying LDL oxidation, it is likely that two mechanisms may contribute: as described for HDL oxidation, the degranulation of phagocytes increases the level of oxygen radicals and of MPO activity, along with a reduction of Paraoxonase 1 (PON1) enzymatic function in HDL-C [43, 51]. PON1, an HDL-associated hydrolytic enzyme with a wide range of substrates, is responsible for most of the antioxidant properties of HDL and has a high capability of protecting against lipid oxidation in inflammation as well as in normal physiologic situations [52–54]. In addition, PON1 has a protective role in coronary artery disease and ischemic stroke [55]. Since several studies indicate that PON1 activity is reduced in inflammatory chronic diseases such as RA [56, 57], it is likely that a decrease in PON1 function can explain the atherogenic lipid profile observed when our patients display a high-disease activity and therefore the increased risk for CVE [58].

The longitudinal design of our study allowed us to clarify whether the effect of different drugs on lipid levels is related to the improvement of disease activity or there is a specific effect of these drugs. This question was raised for different drugs in several clinical trials [39, 59, 60]. Our data suggest that methotrexate, abatacept, and probably leflunomide may exert a beneficial effect upon HDL-C levels that would be independent of the improvement of disease activity, although the size of the effect of these treatments seems to be low.

Our study has some limitations. First, the serum lipid profile of patients included in this study was not available in all the visits of all patients. This is a common drawback in observational retrospective studies, but we think that it could be compensated by the high number of patients and visits carried out, which allowed us to implement longitudinal multivariate analysis nested by the patient and visit with more than 950 visits for the different fractions and more than 1400 visits for TC. The only exception was oxLDL-C analysis, which included 259 visits in 157 patients. This was the consequence of the second limitation of our study, measuring oxLDL-C levels in frozen serum samples. Most kits for measurement of oxLDL, including that from Mercodia® used in this study, recommend using fresh serum samples. However, due to the retrospective nature of our study, we had to use frozen serum samples limiting the number of samples as it was described in the “Patients and methods” section and requiring adjusting the analysis for the time of frozen storage. Nevertheless, despite this limitation, we think that the analysis of oxLDL-C in our study provides interesting information.

Another potential limitation refers to the analysis of the effect of biologic therapies on the lipid profile. Since we studied mainly the first 5 years of follow-up of patients with EA, few visits included treatment with such drugs. The best represented was the TNF inhibitors group (Supplementary Table 1), but the information provided in this study on the effect of abatacept on HDL-C levels relies on information from few visits and it has to be confirmed in a specific study. However, it fits well with a recent study suggesting that abatacept reduces CV risk by 20% more than TNF inhibitors [61].

## Conclusions

Overall, our data suggest that mainly patients that remain with moderate and high-disease activity suffer an additional risk of CV disease due to RA, since only these patients show relevant changes in the levels of HDL-C fraction and oxLDL-C. In addition, the changes in oxLDL-C are especially relevant in patients with low LDL-C. These findings are in keeping with descriptions showing a decreased incidence of CV disease in patients treated either with methotrexate or biologic agents [62–64], since an increasing number of patients reach low-disease activity and remission at present [65]. Moreover, our findings can also explain why a beneficial effect preventing cardiovascular events has not been observed with methotrexate in the general population [66] or with atorvastatin in non-selected RA patients [67].

## Supplementary information

**Additional file 1: Supplementary Figure 1.** Effect of time of frozen serum storage on the measurement of oxidized low density lipoproteins from patients with early arthritis. Data are shown as the individual values of 104 serum samples with a range of frozen storage from 6 to 40 months (black dots) and the linear regression (black line) estimated through the *twoway lfit* command of Stata 12.1.

**Additional file 2: Supplementary Figure 2.** Comparative effect of the different components of disease activity indexes on the total cholesterol levels. A) Total cholesterol. B) LDL cholesterol. C) HDL cholesterol. Data are shown as the standardized coefficients (black dots) and their respective 95% confidence intervals (black lines) from the multivariate analysis described in Methods in which the variable disease activity was substituted by the different components included in the disease activity indexes: global disease assessment by patient (GDA Pat), tender joint count (TJC), swollen joint count (SJC) or C-reactive protein (CRP).

**Additional file 3:** **Supplementary Table 1.** Treatment prescribed to PEARL patients along the follow-up. **Supplementary Table 2.** Sensitivity analysis of variables that have influence on lipid profile only including patients that did not take statins. **Supplementary Table 3.** Variables that have influence on lipid profile, including disease activity estimated by CRP-DAS28. **Supplementary Table 4.** Variables that have influence on lipid profile, including disease activity, estimated with ESR-DAS28**. Supplementary Table 5.** Variables that have influence on lipid profile, including disease activity, estimated with SDAI. **Supplementary Table 6.** Variables that have influence on lipid profile, including disease activity, estimated with CDAI. **Supplementary Table 7.** Variables that have influence on VLDL Cholesterol levels. **Supplementary Table 8.** Variables that have influence on triglyceride levels.

**Additional file 4.** Raw data used for this work (in Stata format).

## Data Availability

All data generated or analyzed during this study are included in the Supplementary File: raw_data.dta If needed, additional information could be available from the corresponding author on reasonable request.

## Abbreviations

ACPA: Anti-citrullinated protein antibodies
ACR: American College of Rheumatology
ApoA1: Apoprotein A1
β coeff.: Beta coefficient
BMI: Body mass index
CV: Cardiovascular
CVE: Cardiovascular event
CDAI: Clinical Disease Activity Index
CRP: C-reactive protein
DAS28: Disease Activity Score (28 joints count)
EA: Early arthritis
ESR: Erythrocyte sedimentation rate
HAQ: Health Assessment Questionnaire
HDL: High-density lipoproteins
HUPI: Hospital Universitario La Princesa Index
IQR: Interquartile range
LDL: Low-density lipoproteins
MTX: Methotrexate
MPO: Myeloperoxidase
N: Number
N: No
n.i.: Not included
oxLDL-C: Oxidized LDL cholesterol
p: *p* value
PEARL: Princesa Early Arthritis Register Longitudinal
PON1: Paraoxonase 1
RF: Rheumatoid factor
Ref.: Reference variable
SD: Standard deviation
SDAI: Simplified Disease Activity Index
s.e.: Standard error
sqrt: Square root
TC: Total cholesterol
TG: Triglycerides
TNF: Tumor necrosis factor
TNFi: TNF inhibitors
UA: Undifferentiated arthritis
VLDL: Very low-density lipoproteins
wk: Week
Y: Yes
