# Supplementary figures and images for: Effects of disease activity on lipoprotein levels in patients with early arthritis: can oxidized LDL cholesterol explain the lipid paradox theory?
Source: Arthritis Res Ther. 2020 Sep 11;22:213. doi: 10.1186/s13075-020-02307-8 (PMC7488761; doi:10.1186/s13075-020-02307-8)

## Slide 1
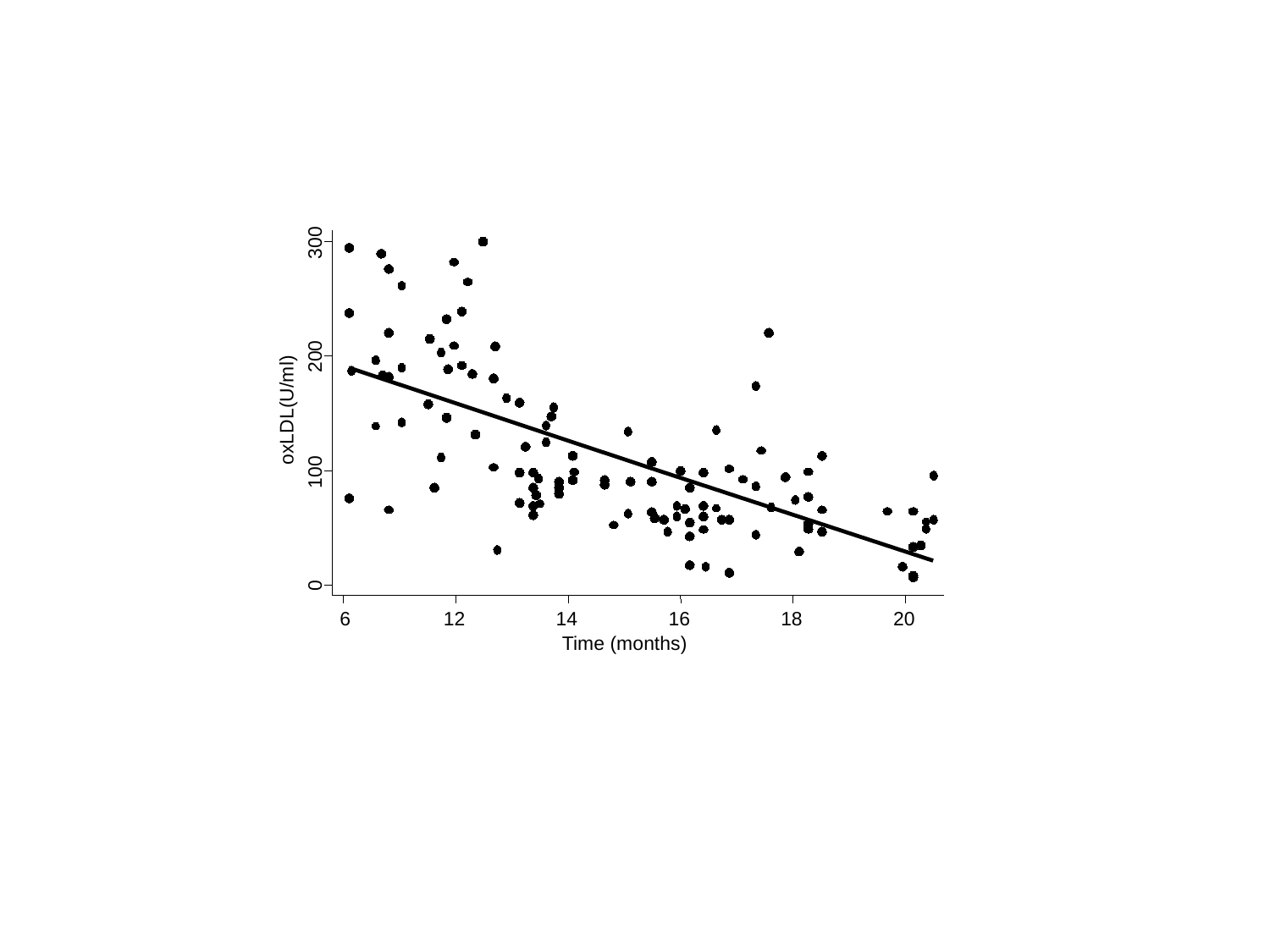

300
200
oxLDL(U/ml)
100
0
6
12
14
16
18
20
Time (months)

Supplement: Supplementary file 1 — Additional file 1: Supplementary Figure 1. Effect of time of frozen serum storage on the measurement of oxidized low density lipoproteins from patients with early arthritis. Data are shown as the individual values of 104 serum samples with a range of frozen storage from 6 to 40 months (black dots) and the linear regression (black line) estimated through the twoway lfit command of Stata 12.1. [file 13075_2020_2307_MOESM1_ESM.pptx]

## Slide 1
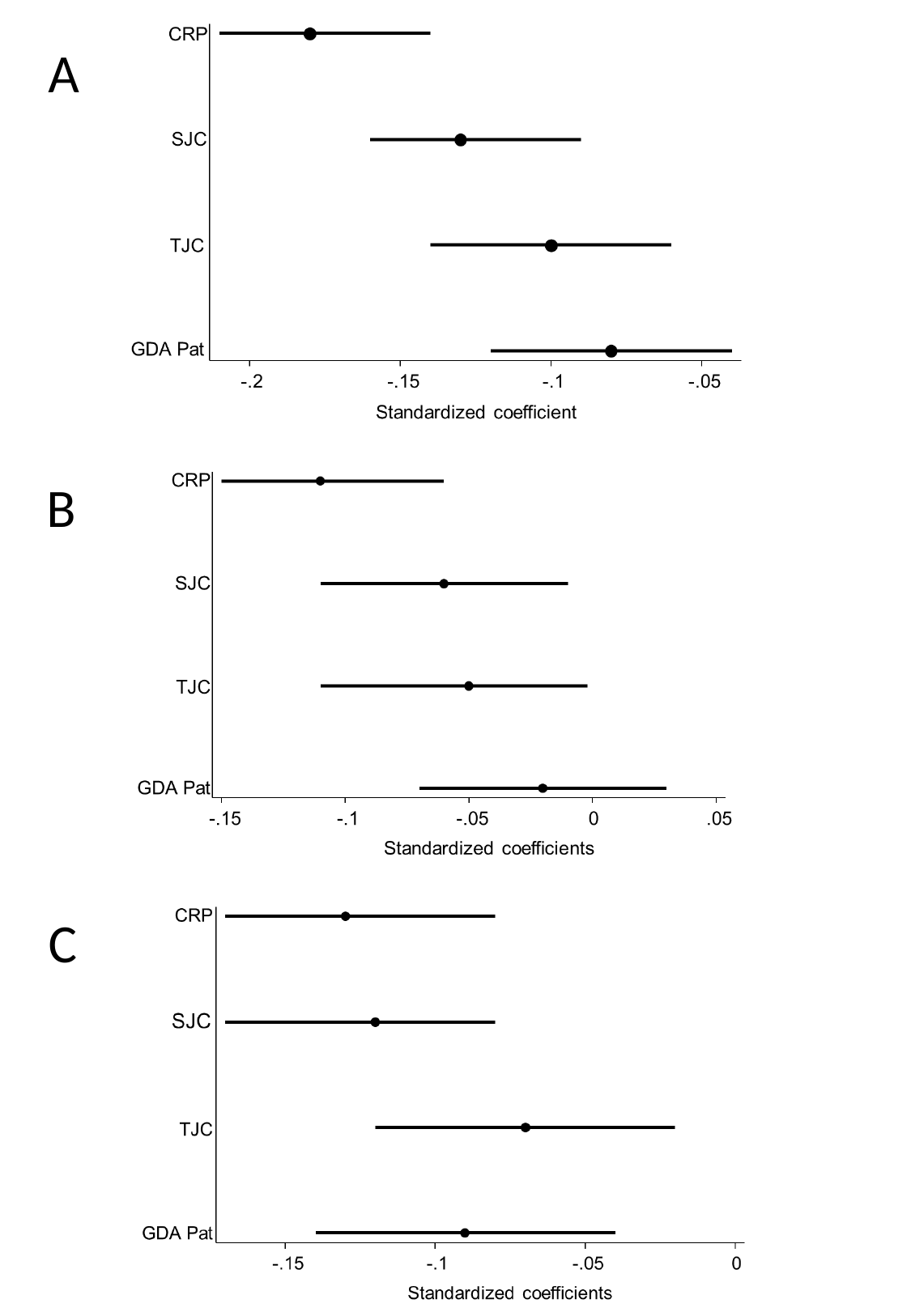

A
B
C

Supplement: Supplementary file 2 — Additional file 2: Supplementary Figure 2. Comparative effect of the different components of disease activity indexes on the total cholesterol levels. A) Total cholesterol. B) LDL cholesterol. C) HDL cholesterol. Data are shown as the standardized coefficients (black dots) and their respective 95% confidence intervals (black lines) from the multivariate analysis described in Methods in which the variable disease activity was substituted by the different components included in the disease activity indexes: global disease assessment by patient (GDA Pat), tender joint count (TJC), swollen joint count (SJC) or C-reactive protein (CRP). [file 13075_2020_2307_MOESM2_ESM.pptx]
