# Supplementary material for: Effects of disease activity on lipoprotein levels in patients with early arthritis: can oxidized LDL cholesterol explain the lipid paradox theory?
Source: Arthritis Res Ther. 2020 Sep 11;22:213. doi: 10.1186/s13075-020-02307-8 (PMC7488761; doi:10.1186/s13075-020-02307-8)
Supplement: Supplementary file 3 — Additional file 3: Supplementary Table 1. Treatment prescribed to PEARL patients along the follow-up. Supplementary Table 2. Sensitivity analysis of variables that have influence on lipid profile only including patients that did not take statins. Supplementary Table 3. Variables that have influence on lipid profile, including disease activity estimated by CRP-DAS28. Supplementary Table 4. Variables that have influence on lipid profile, including disease activity, estimated with ESR-DAS28. Supplementary Table 5. Variables that have influence on lipid profile, including disease activity, estimated with SDAI. Supplementary Table 6. Variables that have influence on lipid profile, including disease activity, estimated with CDAI. Supplementary Table 7. Variables that have influence on VLDL Cholesterol levels. Supplementary Table 8. Variables that have influence on triglyceride levels. [file 13075_2020_2307_MOESM3_ESM.docx]

**Supplementary Table 1.** Treatment prescribed to PEARL patients along the follow-up.

|  | **V2 (6 months)** | | | | **V3 (12 months)** | | | | **V4 (24 months)** | | | | **V5 (60 months)** | | | |
| --- | --- | --- | --- | --- | --- | --- | --- | --- | --- | --- | --- | --- | --- | --- | --- | --- |
|  | N | oxLDL measurement | | p | N | oxLDL measurement | | p | N | oxLDL measurement | | p |  | oxLDL measurement | |  |
|  |  | No (%) | Yes (%) |  |  | No  (%) | Yes (%) |  |  | No  (%) | Yes (%) |  | N | No  (%) | Yes (%) | p |
| MTX | 264 | 73.2 | 77.9 | 0.274 | 261 | 71.4 | 66.9 | 0.357 | 261 | 69 | 60.3 | 0.073 | 133 | 68.6 | 59.4 | 0.167 |
| LEF | 36 | 10.4 | 8.6 | 0.566 | 65 | 21.6 | 10.8 | **0.007** | 84 | 21.9 | 19.9 | 0.628 | 58 | 26.5 | 29.2 | 0.656 |
| AM | 56 | 13.4 | 17.9 | 0.247 | 63 | 14.1 | 20.9 | 0.083 | 61 | 13.6 | 17.9 | 0.244 | 34 | 17.6 | 15.1 | 0.619 |
| SSZ | 12 | 2.6 | 4.3 | 0.373 | 16 | 3.1 | 6.1 | 0.160 | 16 | 2.1 | 7.0 | **0.013** | 9 | 0.9 | 7.5 | **0.020** |
| TNFi | 4 | 0.9 | 1.4 | 0.611 | 12 | 4.4 | 1.3 | 0.101 | 25 | 7.4 | 4.5 | 0.236 | 28 | 10.8 | 16.0 | 0.267 |
| TCZ |  | | | |  | | | | 2 | 0.8 | 0 | 0.255 | 5 | 4.9 | 0 | 0.021 |
| ABA |  | | | | 2 | 0.9 | 0 | 0.252 | 2 | 0.8 | 0 | 0.255 | 2 | 1.9 | 0 | 0.147 |
| RTX |  | | | | 1 | 0.4 | 0 | 0.419 | 4 | 1.6 | 0 | 0.107 | 7 | 5.9 | 0.9 | 0.048 |

Abbreviations: ABA: abatacept; AM: antimalarials; LEF: leflunomide; MTX: metotrexate; oxLDL: oxidized LDL; RTX: rituximab; SSZ: sulfasalazine; TCZ: tocilizumab; TNFi: TNF inhibitors; V: visit

**Supplementary table 2.** Sensitivity analysis of variables that have influence on lipid profile only including patients that did not take statins

|  | Total Cholesterol (mg/dl) | | HDL Cholesterol (mg/dl) | | LDL Cholesterol (mg/dl) | | oxLDL (sqrt-U/ml) | |  |
| --- | --- | --- | --- | --- | --- | --- | --- | --- | --- |
|  | β coefficient ± s.e. | p | β coefficient ± s.e. | p | β coefficient ± s.e. | p | β coefficient ± s.e. | p | |
| Gender  Male  Female | Reference  12.66 ± 3.51 | -  <0.001 | Reference  12.34 ± 2.04 | -  <0.001 | n.i. | - | Reference  -0.87 ± 0.55 | -  0.117 | |
| Age  <45 y  45 to 65 y  > 65 y | Reference  22.58 ± 3.25  24.48 ± 3.72 | -  <0.001  <0.001 | Reference  1.84 ± 1.86  5.57± 2.16 | -  0.322  0.010 | Reference  16.05 ± 3.21  12.82 ± 3.65 | -  <0.001  <0.001 | n.r. | - | |
| BMI | 0.66 ± 0.29 | 0.023 | -0.66 ± 0.17 | <0.001 | 0.71 ± 0.30 | 0.017 | n.i. | | |
| Disease activity  Remission | Reference | - | Reference | - | Reference | - | Reference | - | |
| Low | 0.46 ± 1.76 | 0.793 | -0.97 ± 1.00 | 0.336 | 0.89 ± 2.08 | 0.670 | 0.24 ± 0.53 | 0.654 | |
| Moderate | -4.14 ± 1.99 | 0.030 | -4.16 ± 1.10 | <0.001 | 1.33 ± 2.24 | 0.551 | 0.22 ± 0.53 | 0.671 | |
| High | -19.26 ± 2.61 | <0.001 | -10.10 ± 1.56 | <0.001 | -9.35 ± 2.96 | 0.002 | 1.24 ± 0.75 | 0.096 | |
| Smoker | n.i. | | n.r. | - | n.r. | - | n.i. | | |
| MTX (by mg/wk) | n.r. | - | 2.04 ± 0.91 | 0.025 | n.r. | - | n.r. | - | |
| Leflunomide (by mg/d) | 7.85 ± 2.25 | <0.001 | 1.27 ± 1.25 | 0.308 | 5.15 ± 2.55 | 0.043 | n.r. | - | |
| Abatacept (Y/N) | 48.22 ± 19.65 | 0.014 | 47.42 ± 11.12 | < 0.001 | n.i. | n.i. | n.r. | - | |
| TNFi (Y/N) | n.r. | - | n.r. | - | n.r. | - | 1.50 ± 1.03 | 0.144 | |
| LDL (mg/dl) total | n.i. | | n.i. | | n.i. | | 0.013 ± 0.006 | 0.032 | |
| Time of frozen storage (by month) | n.i. | | n.i. | | n.i. | | -0.004± 0.001 | <0.001 | |

Abbreviations: BMI: body mass index; CDAI: Clinical Disease Activity Index; CRP: C Reactive Protein; d:day; DAS28: Disease Activity Score (counting 28 joints); HAQ: Health Assessment Questionnaire; HUPI: Hospital Universitario Princesa Index; MTX: methotrexate; N: no; Ref.: reference variable; SDAI: Simplified Disease Activity Index; n.i.: not included (not relevant to the model); n.r: not relevant; TNFi: TNF inhibitors; w: week; Y: yes.

**Supplementary table 3.** Variables that have influence on lipid profile, including disease activity estimated by CRP-DAS28.

|  | **Total cholesterol**  **(mg/dl)** | | **HDL cholesterol**  **(mg/dl)** | | **LDL cholesterol**  **(mg/dl)** | | **oxLDL**  **(sqrt-U/ml)** | | |
| --- | --- | --- | --- | --- | --- | --- | --- | --- | --- |
|  | β coeff. ± s.e. | p | β coef. ± s.e. | p | β coef. ± s.e. | p | β coef. ± s.e. | | p |
| Gender  Male  Female | Reference  12.0 ± 3.4 | -  <0.001 | Reference  12.4 ± 1.9 | -  <0.001 | n.i. | - | Reference  -1.058 ± 0.485 | | -  0.030 |
| Age  < 45 years  45-65 years  > 65 years | Reference  23.7 ± 3.3  24.6 ± 3.7 | -  <0.001  <0.001 | Ref.  2.2 ± 1.9  5.5± 2.1 | -  0.245  0.01 | Reference  17.3 ± 3.3  13.1 ± 3.7 | -  <0.001  <0.001 | n.r. | | - |
| BMI | 0.79 ± 0.29 | 0.006 | -0.59 ± 0.17 | <0.001 | 0.73 ± 0.29 | 0.013 | n.i. | | |
| DAS28 (CRP) Remission | Ref. | - | Ref. |  | Reference | - | Reference | - | |
| Low | -0.91 ± 1.66 | 0.582 | -1.34 ± 0.96 | 0.163 | 1.82 ± 1.98 | 0.358 | 0.04 ± 0.45 | 0.921 | |
| Moderate | -5.88 ± 2.24 | 0.009 | -6.32 ± 1.37 | <0.001 | 0.90 ± 2.71 | 0.738 | 0.37 ± 0.55 | 0.507 | |
| High | -15.50 ± 2.65 | < 0.001 | -8.29 ± 1.62 | <0.001 | -6.03 ± 3.04 | 0.047 | 1.33 ± 0.67 | 0.050 | |
| Statins | -27.5 ± 3.1 | <0.001 | n.r. | - | -28.7 ± 3.2 | <0.001 | n.i. | | |
| Smoking | n.i. | | n.r. | - | 7.0 ± 4.5 | 0.118 | n.i. | | |
| MTX (by mg/wk) | n.r. | - | 2.17 ± 0.98 | 0.026 | n.r. | - | n.r. | - | |
| Leflunomide  (by mg/d) | 6.4 ± 2.2 | 0.004 | 2.12 ± 1.29 | 0.101 | 4.89 ± 2.62 | 0.063 | n.r. | - | |
| Antimalarials  (by mg/d) | -0.03 ± 0.01 | 0.068 | 0.01 ± 0.01 | 0.139 | -0.05 ± 0.02 | 0.017 | n.r. | - | |
| Abatacept (Y/N) | 36.73 ± 16.25 | 0.014 | 43.19 ± 9.67 | < 0.001 | n.i. | n.i. | n.i. | n.i. | |
| TNFi (Y/N) | n.r. | - | n.r. | - | n.r. | - | 2.019 ± 1.138 | 0.076 | |
| LDL (mg/dl) | n.i. | | n.i. | | n.i. | | 0.015 ± 0.005 | 0.006 | |
| Frozen storage (by month) | n.i. | | n.i. | | n.i. | | -0.004 ± 0.001 | <0.001 | |

Abbreviations: β coeff.: β coefficient; BMI: body mass index; CRP: C Reactive Protein; d: day; DAS28: Disease Activity Score (counting 28 joints); HAQ: Health Assessment Questionnaire; HDL: high density lipoproteins; LDL: low density lipoproteins; MTX: methotrexate; N: no; oxLDL: oxidized LDL; Ref.: reference variable s.e.: standard error; n.i.: not included (not relevant to the model); n.r: not relevant; TNFi: TNF inhibitors; w: week; Y: yes.

**Supplementary table 4.** Variables that have influence on lipid profile, including disease activity, estimated with ESR-DAS28

|  | Total Cholesterol (mg/dl) | | HDL Cholesterol (mg/dl) | | LDL Cholesterol (mg/dl) | | oxLDL (sqrt-U/ml) | |
| --- | --- | --- | --- | --- | --- | --- | --- | --- |
|  | β coefficient ± s.e. | p | β coefficient ± s.e. | p | β coefficient ± s.e. | p | β coefficient ± s.e. | p |
| Gender  Male  Female | Reference  11.33 ± 3.49 | -  0.001 | Reference  12.78 ± 1.97 | -  <0.001 | n.i. | - | Reference  -1.02 ± 0.49 | -  0.039 |
| Age  <45 y  45 to 65 y  > 65 y | Reference  23.31 ± 3.41  23.96 ± 3.77 | -  <0.001  <0.001 | Reference  1.96 ± 1.92  5.65± 2.12 | -  0.308  0.008 | Reference  16.35 ± 3.35  12.19 ± 3.68 | -  <0.001  0.001 | n.r. | - |
| BMI | 0.62 ± 0.29 | 0.036 | -0.62 ± 0.17 | <0.001 | 0.55 ± 0.29 | 0.061 | n.i. | |
| DAS28  Remission | Reference | - | Reference | - | Reference | - | Reference | - |
| Low | -0.25 ± 2.06 | 0.905 | -1.37 ± 1.15 | 0.233 | -0.66 ± 2.43 | 0.784 | -0.34 ± 0.58 | 0.556 |
| Moderate | -2.46 ±1.76 | 0.161 | -2.74 ± 1.02 | 0.007 | 1.53 ± 2.09 | 0.465 | 0.21 ± 0.47 | 0.648 |
| High | -13.48 ± 2.40 | < 0.001 | -8.45 ± 1.46 | <0.001 | -6.40 ± 2.75 | 0.020 | 0.77 ± 0.60 | 0.202 |
| Statins | -27.47 ± 3.09 | <0.001 | n.r. | - | -28.13 ± 3.19 | <0.001 | n.i. | |
| Smoker | n.i. | | n.r. | - | n.r. | - | n.i. | |
| MTX (by mg/wk) | n.r. | - | 2.08 ± 0.93 | 0.025 | n.r. | - | n.r. | - |
| Leflunomide (by mg/d) | 6.79 ± 2.20 | 0.002 | 2.42 ± 1.24 | 0.051 | 4.44 ± 2.54 | 0.081 | n.r. | - |
| Abatacept (Y/N) | 41.20 ± 17.04 | 0.016 | 40.61 ± 9.29 | <0.001 | n.i. | n.i. | n.r. | - |
| TNFi (Y/N) | n.r. | - | n.r. | - | n.r. | - | 1.56 ± 1.01 | 0.122 |
| LDL (mg/dl) total | n.i. | | n.i. | | n.i. | | 0.012 ± 0.006 | 0.042 |
| Frozen storage (by month) | n.i. | | n.i. | | n.i. | | -0.004 ± 0.001 | <0.001 |

Abbreviations: BMI: body mass index; CDAI: Clinical Disease Activity Index; CRP: C Reactive Protein; d:day; DAS28: Disease Activity Score (counting 28 joints); HAQ: Health Assessment Questionnaire; HUPI: Hospital Universitario Princesa Index; MTX: methotrexate; N: no; Ref.: reference variable; SDAI: Simplified Disease Activity Index; n.i.: not included (not relevant to the model); n.r: not relevant; TNFi: TNF inhibitors; w: week; Y: yes.

**Supplementary table 5.** Variables that have influence on lipid profile, including disease activity, estimated with SDAI

|  | Total Cholesterol (mg/dl) | | HDL Cholesterol (mg/dl) | | LDL Cholesterol (mg/dl) | | oxLDL (sqrt-U/ml) | | |
| --- | --- | --- | --- | --- | --- | --- | --- | --- | --- |
|  | β coefficient ± s.e. | p | β coefficient ± s.e | p | β coefficient ± s.e. | P | β coefficient ± s.e. | | p |
| Gender  Male  Female | Reference  11.51 ± 3.45 | -  0.001 | Reference  12.20 ± 1.99 | -  <0.001 | n.i. | - | Reference  -1.04 ± 0.49 | | -  0.033 |
| Age  <45 y  45 to 65 y  > 65 y | Reference  23.11 ± 3.35  24.24 ± 3.71 | -  <0.001  <0.001 | Reference  2.12 ± 1.91  5.38± 2.13 | -  0.268  0.011 | Reference  16.95 ± 3.31  13.15 ± 3.66 | -  <0.001  0.001 | n.r. | | - |
| BMI | 0.68 ± 0.29 | 0.018 | -0.64 ± 0.16 | <0.001 | 0.66 ± 0.29 | 0.025 | n.i. | | |
| SDAI Remission | Reference | - | Reference | - | Reference | - | Reference | - | |
| Low | 2.41 ± 1.89 | 0.203 | -0.05 ± 1.11 | 0.965 | 3.88 ± 2.28 | 0.089 | -0.44 ± 0.53 | 0.411 | |
| Moderate | -2.11 ± 2.08 | 0.311 | -3.43 ± 1.24 | 0.006 | 3.41 ± 2.51 | 0.174 | -0.15 ± 0.55 | 0.779 | |
| High | -13.22 ± 2.66 | < 0.001 | -7.68 ± 1.64 | <0.001 | -3.58 ± 3.11 | 0.251 | 0.79 ± 0.68 | 0.247 | |
| Statins | -27.03 ± 3.09 | <0.001 | n.r. | - | -28.37 ± 3.25 | <0.001 | n.i. | | |
| Smoker | n.i. | | n.r. | - | n.r. | - | n.i. | | |
| MTX (by mg/wk) | n.r. | - | 2.48 ± 0.97 | 0.011 | n.r. | - | n.r. | - | |
| Leflunomide (by mg/d) | 6.87 ± 2.24 | 0.002 | 2.46 ± 1.29 | 0.0.58 | 5.46 ± 2.62 | 0.037 | n.r. | - | |
| Abatacept (Y/N) | 37.36 ± 16.19 | 0.021 | 40.43 ± 9.69 | <0.001 | n.i. | n.i. | n.r. | - | |
| TNFi (Y/N) | n.r. | - | n.r. | - | n.r. | - | 1.95 ± 1.13 | 0.084 | |
| LDL (mg/dl) total | n.i. | | n.i. | | n.i. | | 0.016 ± 0.005 | 0.005 | |
| Frozen storage (by month) | n.i. | | n.i. | | n.i. | | -0.004 ± 0.001 | <0.001 | |

Abbreviations: BMI: body mass index; CDAI: Clinical Disease Activity Index; CRP: C Reactive Protein; d:day; DAS28: Disease Activity Score (counting 28 joints); HAQ: Health Assessment Questionnaire; HUPI: Hospital Universitario Princesa Index; MTX: methotrexate; N: no; Ref.: reference variable; SDAI: Simplified Disease Activity Index; n.i.: not included (not relevant to the model); n.r: not relevant; TNFi: TNF inhibitors; w: week; Y: yes.

**Supplementary table 6.** Variables that have influence on lipid profile, including disease activity, estimated with CDAI

|  | Total Cholesterol (mg/dl) | | HDL Cholesterol (mg/dl) | | LDL Cholesterol (mg/dl) | | oxLDL (sqrt-U/ml) | |  |
| --- | --- | --- | --- | --- | --- | --- | --- | --- | --- |
|  | β coefficient ± s.e. | p | β coefficient ± s.e. | p | β coefficient ± s.e. | p | β coefficient ± s.e. | p | |
| Gender  Male  Female | Reference  10.63 ± 3.45 | -  0.002 | Reference  12.31 ± 1.94 | -  <0.001 | n.i. | - | Reference  -0.98 ± 0.47 | -  0.035 | |
| Age  <45 y  45 to 65 y  > 65 y | Reference  23.22 ± 3.37  23.04 ± 3.72 | -  <0.001  <0.001 | Reference  2-09 ± 1.89  5.39± 2.09 | -  0.268  0.010 | Reference  16.72 ± 3.36  11.71 ± 3.69 | -  <0.001  0.002 | n.r. | - | |
| BMI | 0.60 ± 0.29 | 0.038 | -0.65 ± 0.16 | <0.001 | 0.66 ± 0.29 | 0.025 | n.i. | | |
| CDAI Remission | Reference | - | Reference | - | Reference | - | Reference | - | |
| Low | 3.03 ± 1.84 | 0.099 | -0.18 ± 1.07 | 0.868 | 3.27 ± 2.20 | 0.138 | -0.18 ± 0.51 | 0.714 | |
| Moderate | -1.90 ± 2.02 | 0.346 | -2.69 ± 1.20 | 0.025 | 2.83 ± 2.42 | 0.242 | 0.01 ± 0.53 | 0.987 | |
| High | -9.16 ± 2.42 | < 0.001 | -6.61 ± 1.53 | <0.001 | -1.60 ± 2.86 | 0.576 | 1.16 ± 0.60 | 0.054 | |
| Statins | -27.71 ± 3.06 | <0.001 | n.r. | - | -29.06 ± 3.21 | <0.001 | n.i. | | |
| Smoker | n.i. | | n.r. | - | n.r. | - | n.i. | | |
| MTX (by mg/wk) | n.r. | - | 2.37 ± 0.95 | 0.013 | n.r. | - | n.r. | - | |
| Leflunomide (by mg/d) | 6.75 ± 2.18 | 0.002 | 2.40. ± 1.26 | 0.0.57 | 5.29 ± 2.54 | 0.038 | n.r. | - | |
| Abatacept (Y/N) | 38.45 ± 16.08 | 0.017 | 40.30 ± 9.62 | < 0.001 | n.i. | n.i. | n.r. | - | |
| TNFi (Y/N) | n.r. | - | n.r. | - | n.r. | - | 1.48 ± 0.99 | 0.137 | |
| LDL (mg/dl) total | n.i. | | n.i. | | n.i. | | 0.016 ± 0.005 | 0.003 | |
| Time of frozen storage (by month) | n.i. | | n.i. | | n.i. | | -0.004± 0.001 | <0.001 | |

Abbreviations: BMI: body mass index; CDAI: Clinical Disease Activity Index; CRP: C Reactive Protein; d:day; DAS28: Disease Activity Score (counting 28 joints); HAQ: Health Assessment Questionnaire; HUPI: Hospital Universitario Princesa Index; MTX: methotrexate; N: no; Ref.: reference variable; SDAI: Simplified Disease Activity Index; n.i.: not included (not relevant to the model); n.r: not relevant; TNFi: TNF inhibitors; w: week; Y: yes.

**Supplementary table 7.** Variables that have influence on VLDL Cholesterol levels.

|  | **DAS28 (CRP)** | | **HUPI** | | **DAS28** | | **SDAI** | | **CDAI** | |
| --- | --- | --- | --- | --- | --- | --- | --- | --- | --- | --- |
|  | β coefficient ± s.e. | p | β coefficient ± s.e. | p | β coefficient ± s.e. | p | β coefficient ± s.e. | p | β coefficient ± s.e. | p |
| Gender  Male  Female | Reference  -2.05 ± 1.30 | -  0.115 | Reference  -2.16 ± 1.29 | -  0.095 | Reference  -2.29 ± 1.30 | -  0.078 | Reference  -2.15 ± 1.31 | -  0.101 | Reference  -2.11 ± 1.29 | -  0.102 |
| Age | n.i. | | n.i | | n.i. | | n.i. | | n.i. | |
| BMI | 0.72 ± 0.12 | < 0.001 | 0.73 ± 0.11 | < 0.001 | 0.72 ± 0.11 | < 0.001 | 0.73 ± 0.12 | < 0.001 | 0.74 ± 0.11 | < 0.001 |
| Remission | Reference | - | Reference | - | Reference | - | Reference | - | Reference | - |
| Low | 0.78 ± 0.81 | 0.338 | 1.35 ± 0.86 | 0.119 | 0.63 ± 1.00 | 0.529 | 0.54 ± 0.90 | 0.547 | 0.51 ± 0.88 | 0.567 |
| Moderate | 1.28 ± 1.15 | 0.269 | 1.29 ± 0.97 | 0.182 | 1.38 ± 0.88 | 0.116 | 0.62 ± 1.03 | 0.547 | 0.23 ± 1.00 | 0.819 |
| High | -0.54 ± 1.25 | 0.665 | -0.65 ± 1.21 | 0.592 | -0.26 ± 1.17 | 0.824 | 0.43 ± 1.27 | 0.733 | 0.65 ± 1.21 | 0.587 |
| Statins | 3.46 ± 1.27 | 0.006 | 3.41 ± 1.25 | 0.006 | 3.44 ± 1.26 | 0.006 | 3.36 ± 1.27 | 0.008 |  | |
| Smoking | n.r. | | n.r. | | n.r. | | n.r. | | n.r. | |
| MTX (by mg/wk) | n.i. | | n.i. | | n.i. | | n.i. | | n.i. | |
| Leflunomide  (by mg/d) | 1.93 ± 1.09 | 0.076 | 1.86 ± 1.07 | 0.081 | 1.81 ± 1.07 | 0.092 | 1.90 ± 1.08 | 0.079 | 1.91 ± 1.06 | 0.072 |
| Antimalarials  (by mg/d) | -2.14 ± 1.38 | 0.120 | -2.10 ± 1.35 | 0.120 | -2.29 ± 1.39 | 0.101 | -1.98 ± 1.38 | 0.151 | -2.06 ± 1.34 | 0.125 |
| TNFi (Y/N) | n.i. | | n.i. | | n.i. | | n.i. | | n.i. | |

Abbreviations: BMI: body mass index; CDAI: Clinical Disease Activity Index; CRP: C Reactive Protein; d:day; DAS28: Disease Activity Score (counting 28 joints); HAQ: Health Assessment Questionnaire; HUPI: Hospital Universitario Princesa Index; MTX: methotrexate; N: no; Ref.: reference variable; SDAI: Simplified Disease Activity Index; n.i.: not included (not relevant to the model); n.r: not relevant; TNFi: TNF inhibitors; w: week; Y: yes.

**Supplementary table 8.** Variables that have influence on triglyceride levels.

|  | **DAS28 (CRP)** | | **HUPI** | | **DAS28** | | | **SDAI** | | | **CDAI** | |
| --- | --- | --- | --- | --- | --- | --- | --- | --- | --- | --- | --- | --- |
|  | β coefficient ± s.e. | p | β coefficient ± s.e. | p | β coefficient ± s.e. | | p | β coefficient ± s.e. | | p | β coefficient ± s.e. | p |
| Gender | n.i. | | n.i. | | n.i. | | | n.i. | | | n.i. | |
| Age  < 45 years  45-65 years  > 65 years | Reference  10.19 ± 7.29  16.82 ± 7.86 | -  0.162  0.032 | Reference  9.58 ± 7.14  17.21 ± 7.69 | -  0.180  0.025 | Reference  9.60 ± 7.26  17.41 ± 7.8 | -  0.186  0.026 | | Reference  9.86 ± 7.31  16.26 ± 7.89 | -  0.178  0.040 | | Reference  10.04 ± 7.14  16.39 ± 7.69 | -  0.160  0.033 |
| BMI | 4.05 ± 0.64 | < 0.001 | 4.10 ± 0.63 | < 0.001 | 4.08 ± 0.64 | < 0.001 | | 4.05 ± 0.64 | <0.001 | | 4.14 ± 0.63 | < 0.001 |
| Remission | Reference | - | Reference | - | Reference | - | | Reference | - | | Reference | - |
| Low | 3.03 ± 3.82 | 0.427 | 7.87 ± 4.08 | 0.054 | 6.17 ± 4.84 | 0.202 | | 6.22 ±4.28 | 0.146 | | 5.45 ± 4.20 | 0.195 |
| Moderate | 5.88 ±5.37 | 0.273 | 7.63 ± 4.55 | 0.093 | 6.36 ± 4.17 | 0.127 | | 5.91 ± 4.76 | 0.214 | | 4.35 ± 4.74 | 0.359 |
| High | -8.97 ± 5.84 | 0.125 | -10.18 ± 5.63 | 0.071 | -4.31 ± 5.44 | 0.429 | | -5.28 ± 5.88 | 0.369 | | -4.83 ± 5.60 | 0.388 |
| Statins | n.i. | | n.i. | | n.i. | | | n.i. | | | n.i. | |
| Smoking | n.r. | | n.r. | | n.r. | | | n.r. | | | n.r. | |
| MTX (by mg/wk) | n.i. | | n.i. | | n.i. | | | n.i. | | | n.i. | |
| Leflunomide  (by mg/d) | n.i. | | n.i. | | n.i. | | | n.i. | | | n.i. | |
| Antimalarials  (by mg/d) | -10.64 ± 6.56 | 0.105 | -10.63 ± 6.46 | 0.100 | -12.31 ±6.73 | 0.067 | | -10.33 ± 6.58 | 0.116 | | -10.92 ± 6.44 | 0.090 |
| TNFi (Y/N) | n.i. | | n.i. | | n.i. | | | n.i. | | | n.i. | |

Abbreviations: BMI: body mass index; CDAI: Clinical Disease Activity Index; CRP: C Reactive Protein; d: day; DAS28: Disease Activity Score (counting 28 joints); HAQ: Health Assessment Questionnaire; HUPI: Hospital Universitario Princesa Index; MTX: methotrexate; N: no; Ref.: reference variable; SDAI: Simplified Disease Activity Index; n.i.: not included (not relevant to the model); n.r: not relevant; TNFi: TNF inhibitors; wk: week; Y: yes
